# Acromial stress fracture in a young wheelchair user with Charcot-Marie-Tooth disease: a case report

**DOI:** 10.1186/1757-1626-1-359

**Published:** 2008-11-28

**Authors:** Randeep S Aujla, Abhinav Gulihar, Grahame JS Taylor

**Affiliations:** 1 Orthopaedic Department, Glenfield Hospital, Groby Road, Leicester, Leicestershire, LE3 9QP, UK

## Abstract

Acromial stress fractures are rare and have not been highlighted as a potential complication of wheelchair use. We report the case of a 22-year old female wheelchair bound patient with Charcot-Marie-Tooth disease who presented with a four-year history of shoulder pain which impaired mobility and quality of life. Plain radiographs showed a cortical irregularity of the acromion but no double-density sign. After CT scans a non-united acromial stress fracture was diagnosed. She had no other shoulder pathology. The new technique of using a superiorly closing wedge osteotomy with cancellous lag screw fixation was successful in correcting the mobile non-united acromial fragment and resolving her pain.

## Background

Scapular fractures make up less than 1% of all fractures with only 9% of these involving the acromion [1]. Most scapular fractures are part of poly-trauma, with 80–90% of cases having associated injuries [2]. Charcot-Marie-Tooth disease is a heterogeneous inherited disorder which causes a lack of proteins in the axon and myelin sheath of neurones. Consequently there is a sensory and motor neuropathy in the limbs, particularly the lower limbs. It is an incurable disease which affects up to 23,000 people in the UK [3]. There has been no reported association of shoulder pathology in sufferers. We describe an atraumatic stress fracture of the acromion occurring in isolation in a young wheelchair user who suffered with Charcot-Marie-Tooth disease.

## Case presentation

Our patient was a 22-year old Caucasian female who worked as a council clerk. She had been wheelchair bound due to Charcot-Marie-Tooth disease for five years and presented with a four-year history of increasing bilateral shoulder pain, with the left being significantly worse than the right. The pain was made worse by fully elevating the arm but there was no reduction in the range of movement. Her wheelchair distance was 200 yards before the shoulder pain prevented her going further. There was a family history of shoulder problems with her Father, who was also wheelchair bound, suffering with a rotator cuff tear. She was a non-drinker and non-smoker with a BMI of 33.

On examination she had wasting of the muscles in the hand with poor strength and coordination in the left arm. There was localised point tenderness over the acromion and to a lesser extent the acromioclavicular joint. Forward flexion of the shoulders was to 180° but this caused pain on the left over the acromion. Glenohumeral abduction was to 90° bilaterally. External rotation was to 70° and internal rotation was to the T10 vertebrae bilaterally. Muscles of the rotator cuff had normal power. There were no clinical signs of impingement, with no low painful arc and negative Hawkins test.

**Figure 1:**
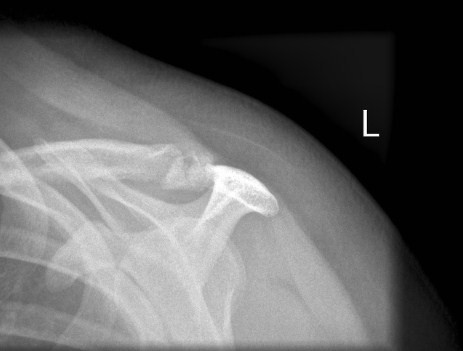
Pre-operative axial radiograph of the left shoulder. It shows an irregularity of the acromion which is poorly visualised. No double density sign is visible.

**Figure 2:**
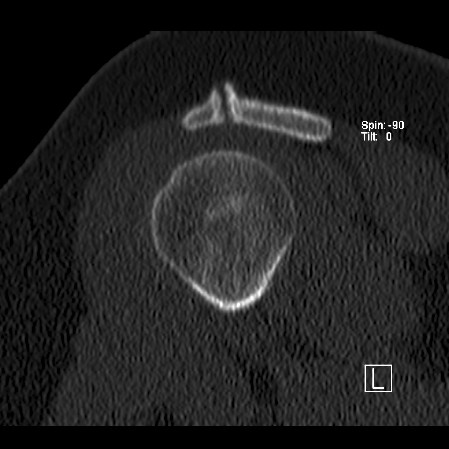
Pre-operative coronal computer tomography image of the left shoulder. It shows the fracture of the acromion with an irregular margin and hypertrophy at the superior aspects of the bony ends. The size of the fragment can also be appreciated.

**Figure 3:**
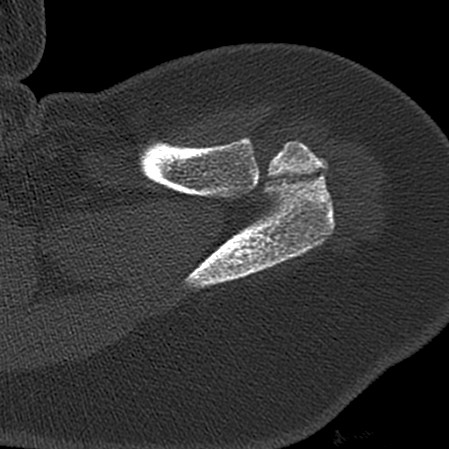
Pre-operative axial computer tomography image of the left shoulder. It shows the ragged bone edges which is indicative of an acromial stress fracture.

Initial radiographs showed an irregularity of the acromion which was considered to be either an os acromiale or an acromial stress fracture (Fig. 1). CT scans showed a 1 cm × 1.8 cm fragment at the anterior aspect of the acromion that had features consistent with a non-united stress fracture of the acromion, such as bony hypertrophy and sharp irregular bone edges (Fig. 2 &3). Rockwood et al described these changes and contrasted them to radiographic features of an os acromial, which would have had rounded uniform cleavage lines [4].

**Figure 4:**
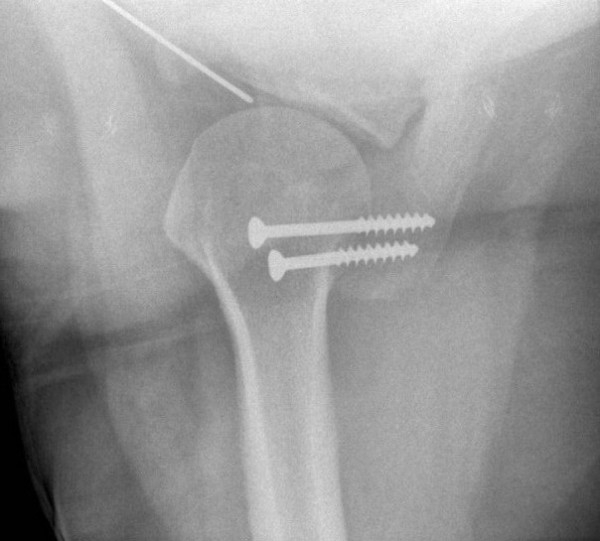
Six weeks post-operative axillary view of the left shoulder. The two lag screws are seen completely within the bone. The fracture has healed with no fracture line visible.

**Figure 5:**
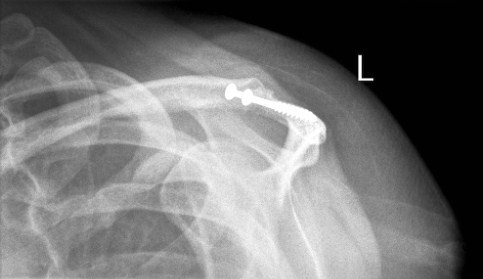
Six weeks post-operative axial view of the left shoulder. The fracture has united with the screws still within the bone.

At time of surgery it was noted that the fragment was mobile and too large to excise. The non-united fracture was then corrected using a superiorly closing wedge excision osteotomy and fixated with two partially threaded cancellous lag screws (Fig. 4 &5). This osteotomy was used to help elevate the anterior acromion and reduce the likelihood of future impingement. A sub-acromial decompression was performed at the same time. The patient remained in a poly-sling for six weeks with only pendulum exercises permitted for the first two weeks. Passive movements were allowed thereafter until union of the fracture at six weeks. After out-patient follow-up at six weeks active movements were initiated. At 6 months follow-up the patient reported no problems, having equal range of movement bilaterally and a pain free left shoulder.

## Discussion

Shoulder pain is reported in 30–40% of wheelchair users. The forces on the shoulder joint are considerable and increase four-fold with rising up an incline and fast wheeling [5]. Numerous pathologies have been linked with wheelchair use, such as rotator cuff impingement, glenohumeral instability, and biceps tendonitis [6].

Clinical history and examination rarely differentiates os acromiale and acromial stress fractures, with both demonstrating point tenderness and pain in the shoulder on full elevation. AP and axillary view shoulder radiographs can reveal a double-density sign or cortical irregularities which are 94.1% sensitive and 100% specific for diagnosis of os acromiale [7]. From our experience small fragments may not be visible on plain radiographs and may require further imaging. Therefore CT scans should be considered in wheelchair users presenting with shoulder pain if plain radiographs reveal little. Cannulated screws with tension band wiring have been proven to be successful in treating os acromiale [8].

Stress fractures are more common in the lower limb. Acromial stress fractures, or symptomatic os acromiale, should be considered in both young and old wheelchair bound patients who present with shoulder pain.

Upon literature review we uncovered eight previous reported cases of acromial stress fracture [2,9-13]. Three occurred in sports athletes, one of which was after sub-acromial decompression surgery [2,10,11]. The remaining five cases were all over 64 years of age, four had associated rotator cuff tears, two were on long-term steroids and one was a wheelchair user [9,12,13].

Our patient was much younger than the other reported cases in non-athletes. She had no associated rotator cuff tear and did not take steroids. Also her bone quality was noted to be good during surgery and it is unlikely a 22-year old would be suffering from osteoporosis, but no formal bone density testing was performed. Her only risk factor was her wheelchair use.

Seven out of the eight reported cases were treated conservatively with immobilisation and physiotherapy. Of these seven, three united, one developed an asymptomatic non-union and three progressed to excision of the fragment with a subsequent good outcome [2,9,10,12,13]. Only one case was managed surgically using two Kirschner wires and a tension band suture [11]. This was performed in a professional tennis player three months after a sub-acromial decompression.

Our presented technique offered a good compression of the osteotomy and a low risk of metal migration which has been reported with use of wires around the shoulder [14]. We appreciate that this technique may not be successful in elderly patients with osteoporotic bone and in these patients surgical excision of the distal fragment may be a better option.

## Conclusion

This case report highlights acromial stress fractures as a potential complication of wheelchair use. The technique of using a superiorly closing wedge osteotomy of the acromion with partially threaded cancellous lag screws to compress the osteotomy led to a successful clinical outcome and has not been previously described. Also the acromial fracture was poorly visualised with standard shoulder radiographs and CT scans provided a clearer diagnosis.

## Abbreviations

BMI: Body mass index; CT: Computerized tomography.

## Consent

Written informed consent was obtained from the patient for publication of this case report and accompanying images. A copy of the written consent is available for review by the Editor-in-Chief of this journal.

## Competing interests

The authors declare that they have no competing interests.

## Authors' contributions

RSA analysed and interpreted the patient history and clinical outcome. He also researched on all aspects of the report and was the main writer of the manuscript. AG and GJT were involved in the clinical management of the patient and were both contributors in writing the manuscript. All authors read and approved the final manuscript.

## References

[B1] Kuhn JE, Blaiser RB, Carpenter JE (1994). Fracture of the acromion process: a proposed classification system. Journal of Orthopaedics and Trauma.

[B2] Hall RJ, Calvert PT (1995). Stress fracture of the acromion: an unusual mechanism and review of the literature. J Bone Joint Surg Br.

[B3] NHS Direct. http://www.nhsdirect.nhs.uk/articles/article.aspx?articleId=441.

[B4] Rockwood CA, Matsen FA, Wirth MA, Lippitt SB (2004). The Shoulder.

[B5] Kulig K, Rao SS, Mulroy SJ (1998). Shoulder kinetic in wheelchair propulsion. Clinical Orthopaedic and Related Research.

[B6] Finley MA, Rodgers MM (2004). Prevalence and identification of shoulder pathology in athletic and non-athletic wheelchair users with shoulder pain. Journal of Rehabilitation Research and Development.

[B7] Lee DH, Lee KH, Lopez-Ben R, Bradley EL (2004). The double-density sign: A radiographic finding suggestive of os acromiale. J Bone Joint Surg Am.

[B8] Warner JJ, Beim GM, Higgins L (1998). The treatment of symptomatic os acromiale. J Bone Joint Surg Am.

[B9] Rask MR, Steinberg LH (1978). Fracture of the acromion caused by muscle forces. A case report. J Bone Joint Surg Am.

[B10] Schils JP, Freed HA, Richmond BJ, Piraino DW, Bergfeld JA, Belhobek GH (1990). Stress fracture of the acromion. American Journal of Roentgenology.

[B11] Rupp S, Seil R, Kohn DM (1998). Surgical reconstruction of a stress fracture of the acromion after arthroscopic after arthroscopic subacromial decompression in an elite tennis player. Journal of Arthroscopic and Related Surgery.

[B12] Dennis DA, Ferlic DC, Clayton ML (1986). Acromion stress fracture associated with rotator cuff arthropathy: A report of three cases. The Journal of Bone and Joint Surgery (American).

[B13] Roy N, Smith MG, Jacobs LGH (2002). Stress fracture of the base of the acromion. Annals of Rheumatic Disease.

[B14] Lyons FA, Rockwood CA (1990). Migration of pins in operations on the shoulder. J Bone Joint Surg Am.

